# Tuberculosis in Wales: Activities of the National Memorial Association

**Published:** 1916-04-01

**Authors:** 


### TUBERCULOSIS IN WALES.

### Activities of the National Memorial Association.

TUBERCULOSIS is much more a social evil than is generally realised, and must be regarded from the broadest epidemiological standpoint. Statistics giving the results of treatment, the incidence of the disease among the persons examined, and the clinical stage which the disease has reached have a certain interest and value, but they have only an indirect bearing on the main problem—namely, the prevention of tuberculosis.

Organisations for the treatment of consumption and, in an unfortunately limited sense, for the prevention of the disease, now exist all over the kingdom; these organisations are steadily acquiring a sound knowledge of etiological factors, and meanwhile preparing the way for important measures by educating the public on the real meaning of tuberculosis as an infectious disease.

The King Edward VII. Welsh National Memorial Association for the prevention and abolition of tuberculosis is a co-ordinated organisation which embraces a large area, including thirteen counties and four county boroughs. It therefore is in a position to gather experience of the ravages of consumption in areas differing widely orographically and in the mode of living of the inhabitants. From the voluminous report of this association tuberculosis officers and other interested persons may expect to glean useful information, or at least to gather impressions of current thoughts and aims in relation to the control of this greatly destructive plague.

The medical aspects of the report summarise the labours and the views of fourteen tuberculosis physicians and of the medical director, Dr. Marcus Paterson. The very large number of statistical tables, which make the report a close rival to the annual volumes of the Metropolitan Asylums Board, do not in the main call for detailed attention except in so far as they are able to show that some progress was made during the year in spite of the serious difficulties which inevitably handicapped the work in Wales, as elsewhere. This third annual report covers the period between April 1, 1914, and March 31, 1915, when 8,868 persons were examined. The first point of interest lies in the fact that of these 40 per cent. were diagnosed as suffering from pulmonary tuberculosis, but in the previous twelvemonth 51 per cent. were so diagnosed; and again in the statement that the cases showing no evidence of active disease amounted to 42 per cent., as against 36 per cent. in the foregoing year. These figures may be taken as indicating that medical practitioners are sending for diagnosis more cases of a doubtful nature than they formerly did—a practice which is to be encouraged as conducing to the discovery of cases of recent infection during the stage when treatment affords hope for good results. The increase in the number of persons found free from tuberculosis is evidence, not of a decrease of work on the part of the physicians, but rather of extra work and increased diagnostic care, for the elimination of the non-tuberculous is a lengthy and difficult task if conscientiously performed.

On the other hand, when the figures showing the classification of pulmonary cases are examined, it is seen that there is a distinct decrease in the numbers referable to the early stages. This may, of course, be set down to the credit of the work of the association by assuming that such cases have been promptly dealt with, and that the supply is becoming limited; but this is a rather rosy view to take. An increase in the advanced types of cases may be explained by the greater facilities for obtaining "hospital" beds and the tendency for practitioners to send severer cases for treatment when they know that institutions are available, and that during their stay therein such patients are at least prevented from spreading infection.

One curious fact emerges from the figures relating to the sex distribution of the patients: there were found suffering from pulmonary tuberculosis only about eighty women for every 100 men, and in the previous year this disparity was even greater. Now, according to the Registrar-General's report, Wales furnishes the highest mortality rate for females in all classes of area for this disease, and this has been the case for many years. In England there is an excess of male over female mortality, especially marked in London and in the South generally. The chief reason to be adduced in explanation of the Welsh figures is the unwillingness of the women to seek medical attention.

To the very important subject of tuberculosis in children many of the tuberculosis physicians make particular reference, and there is one report by Dr. E. H. R. Harries specially devoted to this aspect of the problem. He insists that tuberculosis is a children's disease, but is careful to draw a sharp distinction between the infection and the disease. By the age of about twelve years, we are taught, 90 per cent. of children are infected. In some cases, no doubt, this infection is minimal and of the nature of a protective inoculation. Dr. Harries is probably on the right track when he supposes that a child with tuberculous glands has also, in however mild a degree, a bacillæmia. If a child is infected through the tonsil, a structure rich in blood-vessels, the lymphatics become enlarged and are diagnosed promptly as tuberculous; but it is surely not unreasonable to imagine that some bacilli have escaped the glands and found their way into the blood-stream. The adenitis should be looked upon as an attempt, more or less complete perhaps, but only an attempt, to cope with the invasion of the tubercle bacilli.

Pulmonary disease in children means well-established disease, and does not begin to be common till the third quinquennium. It is amongst children who are no longer infants but too young for school that the beginnings of the disease must be sought for. This is an almost unexplored field, and the children are commonly labelled debilitated. Having eliminated certain conditions, it is not rash to assume the tubercular infection, for tuberculosis must be regarded as a general disease, and localising signs should not be waited for. Of the practical steps to be taken the most frequently recommended is the open-air school with additional nourishment, or removal to a sanatorium with a special section for children. But as the type of case is numerous, and as exclusion from school is in many ways injurious, the solution would seem to lie in the wide provision of open-air schools. These children, as Dr. Harries and others have pointed out, need not be notified as cases of tuberculosis; it is preferable that they should be notified to the school medical officer.

A common difficulty is a lack of facilities for promoting oral hygiene. Dentists are too few and septic mouths are great obstacles in the way of raising the resistance of patients. In not a few cases the benefit accruing after removal of septic stumps or the provision of efficient false teeth must be seen to be believed. Many officers deplore the excessive tea-drinking which seems to be prevalent in Wales, and the frequency with which bread and butter is regarded as the staple fare. Domestic insanitation is another sore point, and with it is allied the "fetish of the unused parlour." Bad housing conditions and domestic insanitation are for the most part found together.

Many somewhat drastic proposals are made. Two observers are so strongly impressed by the importance of the hereditary factor that they suggest legislation to restrict intermarrying amongst collaterals and between notified tubercular persons. The need for power of compulsory removal and detention of advanced "open" cases is also insisted upon; a power which may possibly be granted in the not too distant future. The picture-palace habit is blamed for much direct or indirect infection; while in Swansea there appears to be no abatement of the practice of spitting in public vehicles and in the streets. The careful patient who produces his flask will be shunned, while the user of the dangerous handkerchief or the spitten on the floor is tolerated, even sympathetically.

A word must be said in reference to the Education Campaign, not the least important of the Association's activities. The chief medical lecturer and superintendent is Dr. R. Owen Morris, who is supported by an assistant organiser and two lady lecturers. With the exception of school lectures, which were steadily pursued, the war naturally crippled their activities, but 904 schools were visited, and there were in attendance during the lectures 3,746 adults and 76,538 scholars. Only eighteen public lectures were given. In their plan of campaign are included Press articles, a touring exhibition, a library, and the display at cinemas of slides bearing on the prevention of tuberculosis.

On the lines of a similar effort in the United States it has been proposed to try to institute a Tuberculosis Sunday with the co-operation of clergymen and organisers of brotherhoods, P.S.A.s, and Y.M.C.A.s. The local medical officer of health or other doctor might be induced to address congregations. From the lectures to school-children and the influence of the teachers it is hoped, with some reason, that the hygienic habits of the people may be transformed; for parents are often more ready to be led by their own children than by all the external forces of constituted sanitary authorities.

